# The Effect of Inspiratory Muscle Warm-Up on VO_2_ Kinetics during Submaximal Rowing

**DOI:** 10.3390/sports9030042

**Published:** 2021-03-22

**Authors:** Mati Arend, Jana Kivastik, Jaak Talts, Jarek Mäestu

**Affiliations:** 1 Institute of Sport Sciences and Physiotherapy, Faculty of Medicine, University of Tartu, 51014 Tartu, Estonia; jarek.maestu@ut.ee; 2 Sports Medicine and Rehabilitation Clinic, Tartu University Hospital, 50406 Tartu, Estonia; 3 Institute of Biomedicine and Translational Medicine, Faculty of Medicine, University of Tartu, 50411 Tartu, Estonia; jana.kivastik@ut.ee (J.K.); jaak.talts@ut.ee (J.T.)

**Keywords:** VO_2_ fast and slow component, priming exercise, performance, respiratory muscles

## Abstract

The aim of the study was to investigate the effect of an inspiratory muscle warm-up on the VO_2_ kinetics during submaximal intensity ergometer rowing. Ten competitive male rowers (age 23.1 ± 3.8 years; height 188.1 ± 6.3 cm; body mass 85.6 ± 6.6 kg) took part in this investigation. A submaximal constant intensity (90% P_VO2max_) rowing test to volitional exhaustion was carried out twice with the standard rowing warm-up (Test 1) and with the standard rowing warm-up with additional specific inspiratory muscle warm-up of two sets of 30 repetitions at 40% maximal inspiratory pressure (Test 2). We found a significant correlation between time constant (*τ*_1_) and the VO_2_ value at 400 s in Test 1 (r = 0.78; *p* < 0.05); however, no correlation was found between those parameters in Test 2. In addition, we found a positive association between VO_2max_ from the incremental rowing test and *τ*_1_ from Test 1 (r = 0.71; *p* < 0.05), whereas VO_2_ did not correlate with *τ*_1_ from Test 2. Adding inspiratory muscle warm-up of 40% maximal inspiratory pressure to regular rowing warm-up had no significant effect on oxygen consumption kinetics during submaximal rowing tests.

## 1. Introduction

Rowing is a whole-body sport discipline, which engages a high percentage (70–75%) of active muscle mass that requires large fraction of maximal cardiac output during heavy intensity exercise and stresses inspiratory muscles at high level [1]. Previous research has shown that the fatigue of respiratory muscles may decrease exercise performance [2,3,4,5]. Although respiratory muscles have a unique resilience to moderate exercise intensities, they are susceptible to fatigue when exercising at intensities over 80% of maximal oxygen uptake for prolonged periods of time [6,7]. To delay inspiratory muscle (IM) fatigue, a specific inspiratory muscle training and warm-up (IM-WU) (with 50% and 40% from maximal inspiratory pressure, respectively) has been shown to improve athletic performance in different sport disciplines [8,9,10,11]. However, the mechanisms which contribute to the performance gains after IM training and IM-WU are still unclear [5,9]. One of the proposed mechanisms has been the improvement in oxygen uptake (VO_2_) kinetics [12].

A steady-state VO_2_ is normally reached within 2–3 min during mild to moderate intensity exercise; if exercising at intensities higher than anaerobic threshold, reaching of a steady state VO_2_ is delayed by a supplemental rise in VO_2_ or the slow component of VO_2_ [13,14]. Analyzing VO_2_ kinetics by separating fast and slow components at the onset of exercise may give insight into the factors that could limit exercise performance, as fast increase in VO_2_ at the beginning of intense exercise lowers the use of lactic energy and lactate production [15,16].

During constant work rate, the time constant of the VO_2_ response to a transition from rest to exercise is generally faster in healthy athletes aged 17–23 years compared to non-specifically trained subjects [17,18]. Similarly, a shorter time constant has been linked to improved exercise tolerance and performance in sports such as cycling, running and rowing [19,20,21,22].

Systemic factors such as increased cardio-respiratory work or hormonal changes may contribute to the VO_2_ slow component during higher intensity exercise, but a more important contribution likely originates from the exercising muscles (e.g., changes in fiber recruitment or substrate utilization and increase in muscle temperature or lactic acid concentration) [23]. Numerous interventions have been used to study changes in VO_2_ kinetics and have shown that different types of warm-up substantially reduce the slow component of VO_2_ in the presence or absence of speeded fast component kinetics [18,23,24]. For example, Sahlin et al. [23] pointed out that prior heavy exercise eliminated VO_2_ slow component, and the VO_2_ slow component merged into the initial component of VO_2_ kinetics [23]. A faster VO_2_ kinetic response may be important for performance by reducing the initial oxygen deficit and accumulation of by-products caused by fatigue. For similar work rate, the increase of the initial rate of aerobic energy production would be expected to reduce the depletion of the anaerobic energy reserves [20,25].

Currently, to the best of our knowledge, there are no studies that have investigated the effect of specific inspiratory muscle warm-up on VO_2_ kinetics in rowing exercise, which may give further explanations of the potential associations between the respiratory muscle warm-up and performance. The current study aimed to compare VO_2_ kinetics during high intensity rowing with the traditional rowing warm-up and rowing warm-up together with IM-WU. The hypothesis was that adding IM-WU would reduce the time constant of the fast component and/or the amplitude of the slow component of oxygen consumption kinetics.

## 2. Materials and Methods 

### 2.1. Subjects and Study Design

Ten high level male rowers (Table 1) volunteered to participate in the study. All subjects represented the national team or team candidates and were all healthy for the performance testing. The study was approved by the Research Ethics Committee, and, after the procedures of the study were described, participants gave the written informed consent. The measurements were performed during the preparatory period (i.e., in November), within one month time frame. The subjects were instructed to maintain their regular diet and had to abstain from eating 2 h before testing. The subjects visited the lab three times. To investigate the inspiratory muscle warm-up effect on VO_2_ kinetics, the protocol of two different experimental designs were used. First, the spirometry was measured and maximal inspiratory pressure (MIP) was assessed. Thereafter, the incremental test was performed to measure the individual aerobic power corresponding to VO_2max_ (P_VO2max_) for submaximal exercise protocols. After incremental test, two rowing ergometer tests at 90% of P_VO2max_ were performed in a randomized order using either traditional warm-up (Test 1) or traditional warm-up with the inclusion of inspiratory muscle warm-up (Test 2). The interval between Tests 1 and 2 was 24–48 h. Before the first submaximal test, the subjects had to record the last meal of the day and were asked to consume the identical meal each time before the next testing. The subjects were asked not to participate in any stressful exercise 24 h before the test. No alcohol or caffeinated drinks were allowed before the tests.

### 2.2. Maximal Inspiratory Pressure

To ensure that the subjects had normal respiratory function, spirometry test was carried out. The results from spirometry in all subjects were within normal limits. After spirometry, 15 min of rest was allowed before maximal inspiratory pressure testing (MicroRPM, Micro Medical, Kent, UK) according to the present ATS/ERS statement [26]. After the maneuver was explained, the measurement started from residual volume. For the best result, subjects had to perform 3–5 attempts with differences between the attempts not exceeding 10%. The average of 3 acceptable results was used to calculate the 40% MIP load for the protocol of inspiratory muscle warm-up. All MIP measurements were performed with the subject in a relaxed seated position. The measurement error when testing with Micro RPM has been 2–3% in our lab.

### 2.3. Incremental Exercise Testing

After completing maximal inspiratory testing, the subjects rested for 30 min. After that, the incremental test was started with a 10-min individual warm-up on a rowing ergometer (Concept II, Morrisville, VT, USA) at low intensity (the details are published in [27]). The resistance of the flywheel was set to 5 for all subjects and was kept constant during all tests. The first load was set on 150 W, and the increments were 50 W after every 3 min without rest period [28]. During the test, minute ventilation, maximal oxygen consumption and RER were measured in breath-by-breath mode using a portable oxygen analyzer with facemask (Metamax 3B, Cortex GMBH, Leipzig, Germany). The measurement errors of performing VO_2_ analysis in our lab have been 2–3%. Individual maximal aerobic power (P_VO2max_) was calculated [29]. 

After incremental test, the subjects were familiarized with the PowerBreathe^®^ device (IMT technologies Ltd., Birmingham, UK) to minimize the learning effect during the warm-up.

### 2.4. Submaximal Intensity Rowing Test at 90% P_VO2max_ Intensity

As previously described, the used intensity of the exercise test should be high enough for the possible effect of inspiratory muscle warm-up. The test was performed twice in randomized design to exclude the effect of testing order. Submaximal intensity tests were performed with regular warm-up (Test 1) or with regular warm-up followed by IM-WU (Test 2). During the 90% P_VO2max_ test, the subjects had to exercise as long as possible at the defined intensity but for no longer than 20 min. The display of the ergometer was covered, and the subjects did not see the duration or the distance.

### 2.5. Warm-Up Protocols

A standardized warm-up was performed before Test 1, which consisted of 6 min rowing at 50% P_VO2max_ and 2 min at 75% P_VO2max_ intensity. After 5 min of passive rest, the oxygen mask was applied and the 90% P_VO2max_ test started. Before Test 2, all subjects performed the same standardized rowing warm-up immediately followed by a specific IM-WU with PowerBreathe^®^ device (2 × 30 inspirations at the intensity of 40% of MIP, with 2 min of rest between the sets) [5]. The length of IM-WU was approximately 4.5 min, thus representing the same time period (5 min) that was applied as passive rest in Test 1. To maintain the correct level of inspiratory pressure, the PowerBreathe^®^ device was connected to the manometer to follow the pressure level on the screen.

### 2.6. Modeling of VO_2_ Kinetics

Breath-by-breath VO_2_ data were edited to reduce influence of outliers: each value was compared against the dataset consisting of the preceding and subsequent three data points; the value was excluded, if it was outside 4 SD from the average of this dataset. The remaining VO_2_ data until 400 s of exercise were interpolated to 1 s intervals and then data points were averaged to 5-s intervals to further reduce noise [30].

A non-linear least-squares method was implemented in the MatLab Software (Mathworks, Natick, MA, USA) to fit the VO_2_ data with the model. To characterize the on-transient VO_2_ kinetics, a double-exponential model was used, as follows:

$${\mathrm{VO}}_{2}\left(t\right)=\{\begin{array}{cc} A_{0}+A_{1}\cdot \left(1-e^{-\left(t-TD_{1}\right)/\tau_{1}}\right)\; & for\;t<TD2\\ A_{0}+A_{1}\cdot \left(1-e^{-\left(t-TD_{1}\right)/\tau_{1}}\right)+A_{2}\cdot (1-e^{-\left(t-TD_{2}\right)/\tau_{2}})\; & for\;t\geq TD2 \end{array}$$

where VO_2_(*t*) is the VO_2_ at time *t*; *A*_0_ is the VO_2_ value at rest; and *A*_1_ and *A*_2_, *TD*_1_ and *TD*_2_ and *τ*_1_ and *τ*_2_ are the asymptotic amplitudes, time delays and time constants of the fast and slow VO_2_ components, respectively.

As the warm-up protocol was present 10 min before the start of both tests, we could not measure pure resting VO_2_. Therefore, *A*_0_ as the VO_2_ value at the beginning of the fast phase was used. In the parameter searching process, the data before the beginning of the fast component were ignored; therefore, our analysis excluded the cardio-dynamic phase [15,25,31,32,33].

Identification of the endpoint of the fast phase and determining the characteristics of the VO_2_ slow component was made by consideration of a following collection of constraints:

(1) Parameters *A*_1_, *A*_2_, *TD*_1_, *TD*_2_, *τ*_1_ and *τ*_2_ could not be negative
(2) *τ*_1_ ≥ 10 s
(3) *τ*_2_ ≤ 300 s
(4) *τ*_2_ ≥ 3 · *τ*_1_
(5) 70 ≤ *TD*_2_ ≤ 180

**Figure 1:**
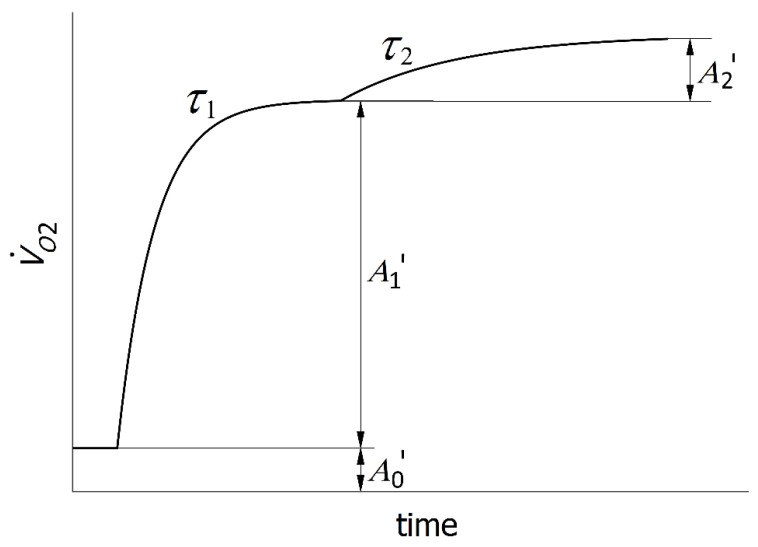
Fast and slow components of VO_2_ kinetics. *A*_0′_ is a calculated value for the resting oxygen consumption; *A*_1′_ and *A*_2′_ are calculated amplitudes for fast and slow phase, respectively; and *τ*_1_ and *τ*_2_ are time constants for the same phases (i.e., the time required to achieve 63% of the amplitude).

Using the equation from Byrne et al. [34], we calculated the value for the resting oxygen consumption (*A*_0′_) for every subject. The value of *A*_0′_ was subtracted from the value of oxygen uptake at the end of the fast component to determine the physiologically relevant amplitude of the fast component (*A*_1′_) (Figure 1). Likewise, the amplitude of the slow component (*A*_2′_) was calculated as a difference between VO_2_ value at *t* = 400 s and VO_2_ value at the beginning of the slow component. *A*_1′_ and *A*_2′_ were presented in preference to the extrapolated asymptotic values.

In most studies [23,32,35], the VO_2_ data from the first 15–20 s at the start of the exercise (i.e., cardio-pulmonary phase when increase in VO_2_ has been attributed to an abrupt increase in pulmonary blood flow) have been excluded from the analysis; therefore, we also decided to exclude all data before the fast exponential increase in the VO_2_ values.

### 2.7. Statistical Analysis

All statistical analyses were performed using version 21 of Statistical Package for Social Sciences (SPSS Inc., Chicago, IL, USA). As data deviated from normality, Wilcoxon signed-rank test was used to compare the difference between the measured parameters from the ergometer test after traditional and IM-WU. The relationships between VO_2_ kinetics parameters and VO_2_ were examined using Spearman’s correlation coefficient. Effect size were calculated as Cohen’s d. Interpretation of the strength of the effect size coefficients is based on guidelines provided by Hopkins: 0–0.09, trivial; 0.10–0.29, small; 0.30–0.49, moderate; 0.50–0.69, large; 0.70–0.89, very large; 0.90–0.99, nearly perfect; and 1.00, perfect [36]. Statistical significance was set at *p* < 0.05 for all the tests.

## 3. Results

The mean (±SD) maximal aerobic power (P_VO2max_) and VO_2max_ from the incremental rowing test are presented in the Table 1.

The subjects did not increase their performance time with the use of additional inspiratory muscle warm-up (828 ± 180 and 840 ± 144 s, respectively). The parameters of the VO_2_ response from the submaximal intensity rowing tests with two different warm-up protocols are reported in Table 2. The VO_2_ data from one subject did not fit the exponential curve for the slow component; therefore, we excluded his data from VO_2_ slow component. No significant differences in any VO_2_ kinetics parameters were found between the two test protocols. For Test 1, the individual ranges for *τ*_1_ and *τ*_2_ were 12.18–30.96 and 53.24–252.64 s, respectively. For Test 2, the individual ranges for *τ*_1_ and *τ*_2_ were 12.13–28.90 and 36.47–299.99 s, respectively. 

Table 3 shows the correlations between different parameters describing the VO_2_ kinetics during two submaximal tests. There was a significant positive correlation between *τ*_1_ and *A*_1′_ (r = 0.85) and between *τ*_1_ and VO_2_ value at 400 s (r = 0.78) in Test 1, but both of those correlations disappeared in Test 2. In both tests, *A*_1′_ was strongly correlated with the VO_2_ value at 400 s (r = 0.91 and r = 0.92, respectively).

**Figure 2:**
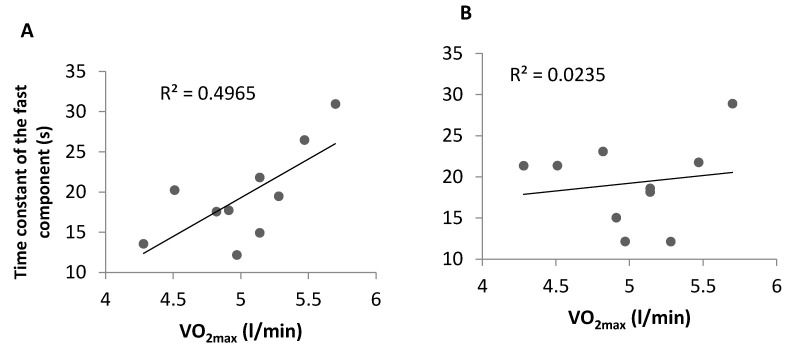
Relationship between maximal oxygen consumption (VO_2max_) and time constant of the fast component (*τ*_1_) during: Test 1 (**A**) (*p* < 0.05); and Test 2 (**B**) (*p* > 0.05).

There was also a significant correlation between VO_2max_ from the incremental rowing test and *τ*_1_ from Test 1 (r = 0.71; *p* < 0.05), whereas VO_2max_ did not correlate with *τ*_1_ from Test 2 (Figure 2).

## 4. Discussion

To our best knowledge, this is the first study to compare oxygen consumption kinetics during high intensity rowing with different warm-up protocols—the first with the regular rowing warm-up and the second with the regular rowing warm-up with the added inspiratory muscle warm-up (IM-WU). The hypothesis of the current study was not supported, as we did not find any significant differences in the fast and slow component VO_2_ kinetics between the two warm-up protocols. 

Performing warm-up before exercise is common in sports, and it is used regularly to influence the physiological response to subsequent exercise [5]. Previous research has shown that the fatigue of the inspiratory muscles (IM) during intense exercise might cause a “steal phenomenon” [6,30] to reduce the blood flow to the exercising limb muscles by means of reflex vasoconstriction. This might affect oxygen uptake negatively and limit exercise tolerance. Several studies have shown that specific inspiratory muscle training or warm-up (IM-WU) can increase the strength of the inspiratory muscles and, therefore, also delay IM fatigue and may have positive effects on sport performance [8,31,37].

Previous studies have reported time constants for the fast phase (*τ*_1_) ranging 35–50 s in general population, approximately 15–30 s in highly trained athletes and 63–75 s in patients with cardiopulmonary disease [15,21,32,33,38,39]. In our study, the mean values for *τ*_1_ (19.50 and 19.26 s for Test 1 and Test 2, respectively) were similar to those reported previously by Ingham [21]; their elite rowers had faster *τ*_1_ than club level rowers for high-intensity exercise (18.7 ± 2.1 and 22.4 ± 3.7 s, respectively). Mean *τ*_1_ values from other studies in rowers have been 16 [15], 26.5 [32], 13.6 [33] and 23 s [38] 

The recruitment of a greater muscle mass could potentially compromise muscle perfusion, particularly during heavy exercise where a larger fraction of the maximal cardiac output is used by active muscles. If muscle perfusion were a limiting factor for VO_2_ kinetics, this would result in longer *τ*_1_ when a greater muscle mass is recruited (e.g., in rowing). However, Roberts et al. [32] showed that pulmonary VO_2_ kinetic responses were similar during moderate and heavy intensity upright cycle and rowing ergometer exercises in physically active men. Koga et al. [40] also reported no significant differences in *τ*_1_ between one- and two-legged cycle ergometry for either moderate or strenuous exercise. If *τ*_1_ is not significantly altered by the recruitment of a higher muscle mass, this could suggest that O_2_ availability does not limit VO_2_ kinetics even during heavy exercise involving a large muscle mass [32]. In the case the workloads were sub-maximal, such as in our tests, cardiac output could be increased during rowing to ensure that muscle perfusion in all areas (i.e., legs, arms and respiratory muscles) was adequate for the better blood flow to the working muscles. This could be the reason adding the IM-WU could not improve the perfusion of exercising muscles and did not change the fast component of the VO_2_ kinetics in our study (both *τ*_1_ and *A*_1′_ were similar in the two tests, Table 2).

It has been suggested that the VO_2_ slow component (*τ*_2_) is primarily linked to the progressive recruitment of motor units with higher order (Type II or fast-twitch) fibers in the exercising muscle [41,42,43]. High-performing rowers should show higher proportion of Type I fibers and, therefore, smaller slow component. However, slow component in elite rowers has been reported greater because of the greater power outputs performed [21]. Training or warm-up could enhance the recruitment of Type I fibers and, therefore, would diminish the slow component and signify an improved exercise economy/efficiency [44,45]. We could not find significant differences in parameters describing the slow component between the two tests in our study. The mean values for *τ*_2_ (105.56 and 101.17 s for Test 1 and Test 2, respectively) were close to those reported previously in rowers (109.6 s) in a study by Demarie et al. [39]. However, there have been quite different *τ*_2_ values in the literature, e.g., in one study, the mean values for *τ*_2_ during high-intensity exercise were 207 s in club level and 242 s in elite rowers [21], whereas, in another study, the mean value for *τ*_2_ was only 48.4 s [42].

Similarly, there are quite different results for the amplitude of the slow component (*A*_2_) in studies of VO_2_ kinetics in rowers [15,21,32,38]. This can mostly be explained by differences in the training status/history, power outputs performed by groups of subjects and therefore differences in the maximal VO_2_ achieved during the test. The mean values for *A*_2_ and VO_2max_ (at 400 s) did not change after adding inspiratory muscle warm-up (from 0.26 to 0.28 L/min and from 4.86 to 4.84 L/min, respectively). In relative terms, the amplitude of the slow phase in current study was quite low: 5.4% of the maximal VO_2_ in Test 1 and 5.8% in Test 2. Other studies with rowers have shown very different maximal VO_2_ values (in the range 3.15–5.09 l/min) and corresponding *A*_2_ values ranging 6.7–10.7% [21,32,39]. As *A*_2_ amplitude is affected by the used intensity, future studies should rather focus on constant, but relatively high submaximal intensities to investigate potential effect of IM-WU. The use of maximal performance test might be complicated due to potentially different pacing strategies that can be used naturally by the subjects. Someone might prefer to do the fast first half and then try to hold the pace as high long as possible, while others might go for the more constant pace strategy. 

Previous studies have found conflicting results regarding the relationship between *τ*_1_ and VO_2max_. Some authors have shown shorter time constant in athletes with higher VO_2max_ [21,38], while others have found no correlation between *τ*_1_ and VO_2max_ [44,46,47]. Interestingly, we observed a significant positive correlation between *τ*_1_ and VO_2max_ from the incremental rowing test and VO_2_ value at 400 s in Test 1_,_ but not in Test 2. Poole et al. [12] suggested a model with two zones demonstrating the effects of altering muscle O_2_ delivery on VO_2_ kinetics: O_2_-delivery-independent zone where decrease in O_2_ delivery does not change the time constant and O_2_-delivery-dependent zone where VO_2_ kinetics become slower (i.e., *τ*_1_ becomes larger) with further reduction in O_2_ delivery. There is an ongoing discussion about whether we can position healthy individuals in a specific place in that model, on either side of the “tipping point” between the two zones, or we have to believe that in a specific exercising subject there may be populations of muscle fibers operating on the right (slow-twitch, non-O_2_-delivery-limited fibers) and some other fibers on the left (fast-twitch, O_2_-delivery-limited fibers) of the tipping point [12,42]. Our study results show that this group of rowers exercised mostly in the O_2_-delivery-independent zone and, therefore, IM warm-up did not change *τ*_1_, and this also caused relatively low slow phase in the group. 

### Limitations

In this study, we used rowing at 90% P_VO2max_ intensity but submaximal intensity rowing test might not offer enough subjective stimuli to prepare or start the respiratory system fast enough to show positive gains in athletic performance. Therefore, testing at higher intensity (e.g., 95–100% VO_2max_) might offer more insight.

Technology to record both respiratory and locomotor (usually leg) muscle oxygenation kinetics using near-infrared spectroscopy (NIRS) are also available and some studies have supported the existence of a competition for oxygen availability between limb and respiratory muscles [48,49], but some did not confirm the respiratory “steal” phenomenon and suggested that the increase in respiratory muscle blood flow might result from other territories than locomotor muscles [50,51]. Unfortunately, we had no possibility to use NIRS to assess changes in O_2_ delivery to exercising muscles.

Comparing the results of our study with those of others is problematic because of the varying modeling techniques. However, the time constants are quite similar to those found by others. Determination of *τ*_1_ with sufficiently high confidence has typically required multiple exercise transitions, limiting its clinical application. Optimizing the signal/noise ratio can reduce the number of transitions necessary for accurate determination of *τ*_1_, potentially enhancing its clinical application [38]. One other negative factor may be the measurement error of the testing equipment since the effect of the warm-up protocols on the VO_2_ kinetics is probably small and therefore within the measurement error. Future studies should take the above-mentioned considerations into account. Furthermore, we suggest using higher intensities for both IM-WU and constant intensity tests. For the latter, the intensity should be higher than anaerobic threshold to induce higher oxygen demand, which further stresses oxygen consumption kinetics at higher rate.

## 5. Conclusions

In this study, the additional inspiratory muscle warm-up of two sets of 30 repetitions with 40% maximal inspiratory pressure to traditional rowing warm-up had no significant effect on oxygen consumption fast or slow component kinetics during 90% P_VO2max_ intensity rowing tests. Therefore, we can conclude that adding the specific inspiration muscle warm-up at the intensity of 40% MIP to traditional rowing warm-up does not have any significant advantage in practice.

## Figures and Tables

**Table 1:** Maximal oxygen consumption and performance parameters of the subjects during the incremental rowing test.

	Subjects (*n* = 10)		
	Mean ± SD	Min	Max
Age (y)	23.1 ± 3.8	20.0	26.0
Height (cm)	188.1 ± 6.3	180.0	202.0
Body mass (kg)	85.6 ± 6.6	70.5	92.0
Rowing experience (y)	8.5 ± 3.2	4.5	12.0
P_VO2max_ (W)	328.7 ± 40.0	275.0	383.0
VO_2max_ (mL·min^−1^·kg^−1^)	50 ± 4.0	43.0	57.0

P_VO2max_, maximal aerobic power; VO_2__max_, maximal oxygen consumption.

**Table 2:** Parameters of the VO_2_ kinetics during two rowing tests at 90% P_VO2max_ with traditional rowing warm-up (Test 1) and with traditional rowing warm-up with specific inspiratory muscle warm-up (Test 2).

	N	Test 1	Test 2	% Change	*p*	Effect Size (Cohen’s d)
*A*_0′_ (L/min)	10	0.26 ± 0.02	0.26 ± 0.02	0	-	-
*τ*_1_ (s)	10	19.50 ± 5.80	19.26 ± 5.20	−1.6%	0.69	0.04
*A*_1′_ (L/min)	10	4.30 ± 0.35	4.28 ± 0.42	−0.5%	0.75	0.05
*TD*_2_ (s)	9	128.32 ± 35.16	125.52 ± 33.18	−2.2%	0.88	0.08
*τ*_2_ (s)	9	105.56 ± 64.00	101.17 ± 61.51	−4.2%	0.83	0.07
*A*_2′_ (L/min)	9	0.26 ± 0.16	0.28 ± 0.17	7.7%	0.83	0.12
VO_2_ at 400 s (L/min)	9	4.86 ± 0.13	4.84 ± 0.14	-0.4%	0.76	0.15

Values are mean ± SD; *A*_0′_, baseline oxygen consumption; *τ*_1_, time constant of the fast component; *A*_1′_, amplitude of the fast component; *TD*_2_, time delay of the slow component; *τ*_2_, time constant of the slow component; *A*_2′_, amplitude of the slow component.

**Table 3:** Correlation coefficients between VO_2_ kinetics parameters.

	Test 1				Test 2			
	*τ*_1_	*τ*_2_	VO_2_ at 400 s	*A*_1′_	*τ*_1_	*τ*_2_	VO_2_ at 400 s	*A*_1′_
*τ*_2_	0.49				0.50			
VO_2_ at 400 s	0.78 *	0.50			0.21	0.04		
*A*_1′_	0.85 **	0.34	0.91 **		0.33	−0.08	0.92 **	
*A*_2′_	−0.09	0.40	0.28	−0.14	−0.53	0.29	0.15	−0.26

*τ*_1_, time constant of the fast component; *A*_1′_, amplitude of the fast component; *τ*_2_, time constant of the slow component; *A*_2′_, amplitude of the slow component. * Correlation is significant at the 0.05 level (two-tailed). ** Correlation is significant at the 0.01 level (two-tailed).

## Data Availability

The data presented in this study are available on request from the corresponding author.
